# Necrobiosis lipoidica: A case report with dermoscopic review

**DOI:** 10.1002/ccr3.3713

**Published:** 2021-01-03

**Authors:** Samir Shrestha, Natalia Spierings, Suchana Marahatta

**Affiliations:** ^1^ BP Koirala Institute of Health Sciences Dharan Nepal; ^2^ Dermatologist London Medical 49 Marylebone High Street London UK

**Keywords:** dermoscopy, diabetes mellitus, granulomatous skin condition, necrobiosis lipoidica

## Abstract

The diagnosis of NL is made traditionally by Histopathological Examination (HPE). This case report will aid for alternative noninvasive modality for confirmation of the condition via dermoscopy and differentiating it from other granulomatous skin condition.

## Graphical abstract

The diagnosis of NL is made traditionally by HPE. This case report will aid for alternative noninvasive modality for confirmation of the condition via dermoscopy and differentiating it from other granulomatous skin condition.

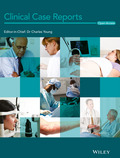

## 1 INTRODUCTION

We present a case of necrobiosis lipoidica in a 55‐year‐old lady. Histopathological examination is used to confirm the diagnosis of NL. Dermoscopy may be used as an alternative tool for confirmation of the diagnosis. In this case report, we present the dermoscopic findings of the condition and review of existing literature.

Necrobiosis lipoidica (NL) is a rare idiopathic, chronic granulomatous skin condition that usually presents as an asymptomatic erythematous papule on the legs progressing to waxy plaque that may develop atrophy and ulcer.^1^ It may present as a complication of diabetes mellitus (DM) in about 0.3% of the patients.^2^ Dermoscopy may be a useful noninvasive tool to differentiate NL from other granulomatous skin conditions like lupus vulgaris (LV), cutaneous leishmaniasis (CL), granuloma annular (GA), and cutaneous sarcoidosis (CS). However, little is known about the characteristic findings of NL on dermoscopy. We present a case of NL with its dermoscopic features and a review of the literature.

## 2 CASE REPORT

**FIGURE 1:**
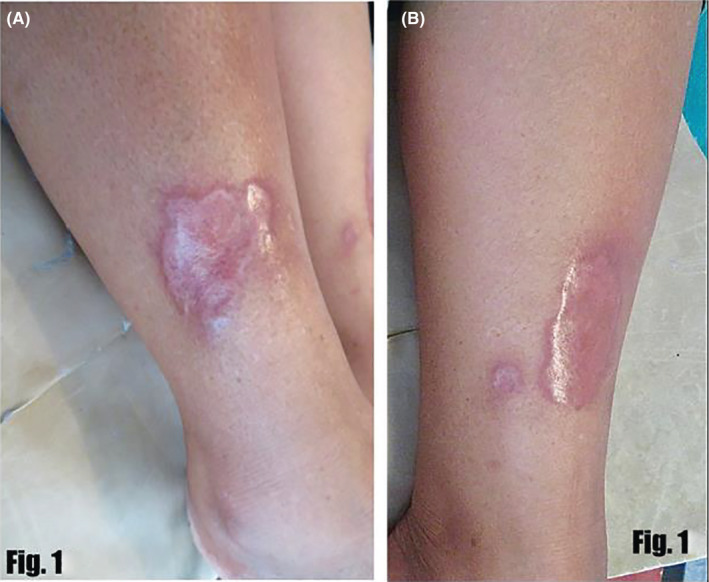
A and B, Showing the waxy erythematous plaques on bilateral shin of the patient

**FIGURE 2:**
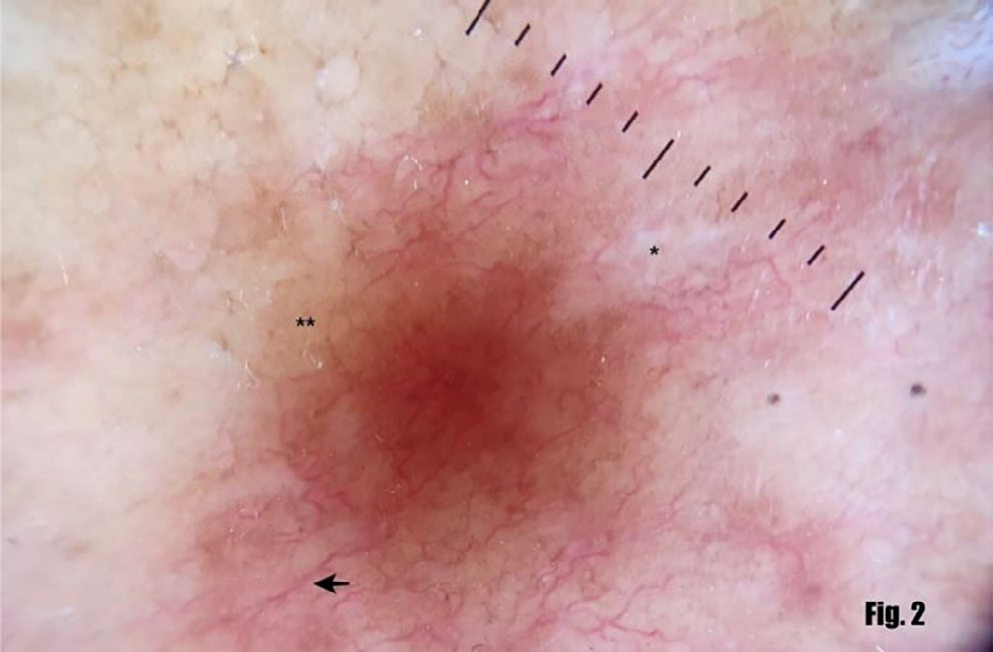
Showing the dermoscopic appearance of the lesion (arrow showing linear vessels with branches distributed uniformly, * white linear streaks, ** yellow structureless area)

A 55‐year‐old lady presented with a 3‐year history of two asymptomatic erythematous pinhead‐sized papules, one on each shin. There was no history of any trauma, DM, or thyroid disorders prior to the appearance of these lesions. The papule on the right leg gradually increased in size over 3 years forming a 5 by 4 cm waxy erythematous irregularly shaped plaque (Figure 1A) with erythematous margin. The center of the plaque comprised telangiectatic vessels with yellow‐hued areas of atrophy. There was no local rise in temperature or tenderness. The papule on the left shin increased in size over the same period forming a similar waxy erythematous round to oval 5 by 2 cm plaque (Figure 1B). On examination at presentation, another plaque with a diameter of about 1.5 cm was found 2 cm medial to the initial lesion on her left shin. Dermoscopic examination (DermLite DL4 with non‐polarizing filter) revealed linear vessels with branches distributed uniformly on the background of yellow structureless areas. White linear streaks were also visible (Figure 2).

**FIGURE 3:**
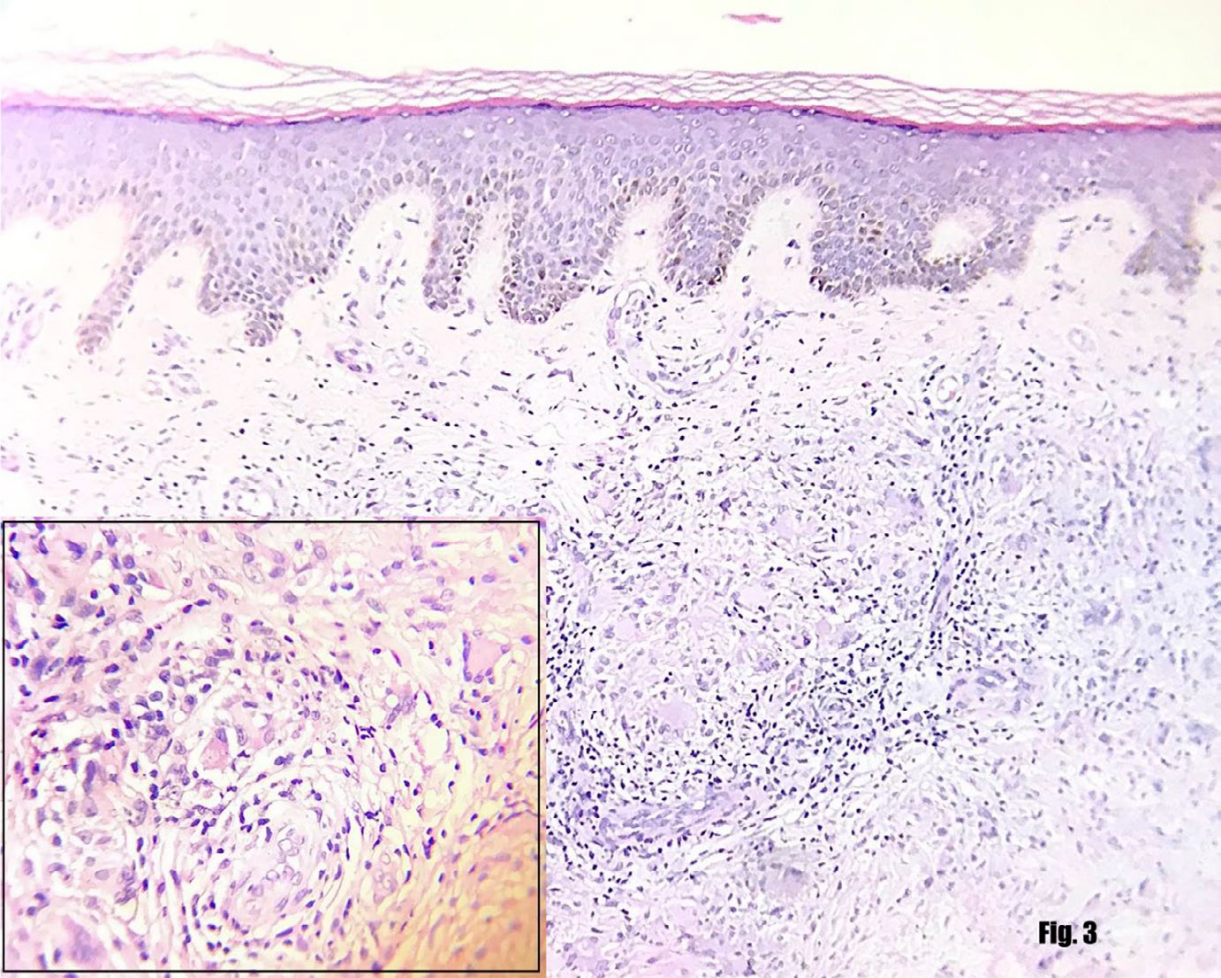
Showing the granulomatous inflammatory infiltrate throughout the dermis surrounding the degenerated collagen. The inflammatory infiltrate comprised of epithelioid histiocytes, lymphocytes, plasma cell, and Langerhans‐type giant cell (shown in inlet within the figure)

Based on the clinical and dermoscopic examination, a provisional diagnosis of NL was made. Blood investigation showed fasting blood sugar of 210 mg/dL, postprandial blood sugar of 300 mg/dL, and HbA1c of 7.3, and thyroid function test was normal. HPE revealed epidermis with basket weave hyperkeratosis and irregular acanthosis. There were multiple areas in dermis with degeneration of collagens surrounded by inflammatory infiltrates. The inflammatory infiltrate comprised of epithelioid histiocytes, lymphocytes, plasma cells, and Langerhans‐type giant cell. Stain for acid‐fast bacilli was negative. The clinical and histological findings confirmed the diagnosis of NL (Figure 3).

The patient was treated with potent topical steroids to apply once daily to the lesions and referred to the internal medicine team for the management of newly diagnosed DM.

## 3 DISCUSSION

Necrobiosis lipoidica is associated with DM, sarcoidosis, thyroid disorders, inflammatory bowel disorder, and healthy individuals.^3^ The association of NL with DM varies from 11% to 87%.^4^, ^5^ Various theories regarding the pathogenesis of the condition have been suggested. For example, in DM, microangiopathy due to the deposition of glycosylated protein may be the cause of the NL. Others have considered hypoxia as the cause of the NL.^6^

Necrobiosis lipoidica presents as painless well‐defined discrete erythematous papules or small plaques or nodules usually on the leg that later coalesce forming a larger waxy, erythematous to yellowish plaque. The center of the plaque may show the areas of atrophy and telangiectasia. Later, the lesion may develop ulceration in around 30% of cases and squamous cell carcinoma in rare cases.^4^

Histopathological Examination (HPE) done in our case showed degeneration of collagen throughout the dermis with palisading granulomas comprising predominantly of lymphocytes and histiocytes that confirmed the diagnosis. The absence of dermal mucin deposition differentiates it from GA.^6^, ^7^

Histopathology is must for the diagnosis of NL. Dermoscopy can also be used as a noninvasive tool to confirm the diagnosis. A few case series and reports highlighted diagnosing and differentiating NL dermoscopically from other granulomatous skin conditions as highlighted in Tables 1 and 2.

**TABLE 1:** Dermoscopy findings of NL^8^, ^9^, ^10^

Characters	Findings	Possible explanation
Morphology of vessel	Linear curved vessels (comma‐shaped) initially, progresses to become linear serpentine and later linear with branches (arborizing vessel).	In the earlier lesion since there is no epidermal changes the comma shaped vessel is due to the dilated vessels of papillary dermis. As the lesion progresses, there will be epidermal atrophy due to which the dilated vessels in deep dermis will be visible that appears as linear serpentine and later linear with branched morphology.
Vessel distribution	Vessels are distributed uniformly.	
Background	Vessels are present on the background of uniformly distributed yellow structureless areas and white linear streaks.	Yellow structureless areas represent dermal granuloma whereas white linear streaks represent fibrosis.
Pigment network	Brown pigmented networks may be visible in advanced lesions	This change is due to the stimulation of melanocytes at the dermo epidermal junction. This finding is nonspecific and is common to many inflammatory skin lesions.

**TABLE 2:** Differentiating dermoscopy findings of NL from other granulomatous conditions^11^, ^12^, ^13^

Dermosopic findings	NL	GA	CS	CL	LV	BCC
Morphology of vessel	Linear vessels with branches. The arborizing vessels in NL are almost equal in diameter without ramification into fine capillaries.	No prominent vessel	Linear vessels that are shorter and less branched than NL	Same as CS	Same as CS	Classical arborizing pattern The vessels ramify into fine capillaries.
White linear streaks	Present	Absent	White reticular lines	White reticular lines	White reticular lines	Absent
Background	Structureless yellow white	Orange red to homogenous white red structureless area at the peripheral margin	Orange globules	Structureless yellow white	Yellow to golden globules	None
Other			Milia like cyst	Milia like cyst	Milia like cyst	Blue and brown pigment structures

Hence, from this report, we would like to highlight that progression of vessel morphology from linear curved (comma‐shaped) to linear serpentine to linear with branches (arborizing vessels) with the progression of the disease, that are distributed uniformly on the structureless yellow‐white background, is an important clue on dermoscopy for diagnosing NL. In the classical arborizing pattern of BCC, the vessels ramify into the finest capillaries. The arborizing vessels in NL are almost equal in diameters without ramification into the finest capillaries and the presence of multiple anastomosing ramifications.

## CONFLICT OF INTEREST

None.

## AUTHOR CONTRIBUTIONS

SS: designed the study, involved in record collection, and wrote the manuscript. NS: edited the manuscript and provided guidance. SM: conceptualized the study, designed the study, edited the manuscript, provided guidance, and approved the final version of the manuscript.

## ETHICAL APPROVAL

Written consent was taken from the patient for the publication of the case report and the images.

## References

[1] Kota S , Kota S , Modi K , Jammula S , Meher L . Necrobiosis lipoidica diabeticorum: a case‐based review of literature. Indian J Endocrinol Metab. 2012;16(4):614. 22837927 10.4103/2230-8210.98023 PMC3401767

[2] Investigation P . Lipoidica. 2015.

[3] Reid SD , Ladizinski B , Lee K , Baibergenova A , Alavi A . Update on necrobiosis lipoidica: a review of etiology, diagnosis, and treatment options. J Am Acad Dermatol [Internet]. 2013;69(5):783‐791. 10.1016/j.jaad.2013.05.034 23969033

[4] Smith JG , Annulare G . Lipoidica. 2015;17‐22.

[5] O'Toole EA , Kennedy U , Nolan JJ , Young MM , Rogers S , Barnes L . Necrobiosis lipoidica: only a minority of patients have diabetes mellitus. Br J Dermatol. 1999;140(2):283‐286. 10233223 10.1046/j.1365-2133.1999.02663.x

[6] Tong LX , Penn L , Meehan SA , Kim RH . Necrobiosis lipoidica. Dermatol Online J. 2018;24(12):6‐9. 30677798

[7] Shulstad R . Granuloma annulare. Adv NPs PAs. 2013;4(11):21‐22. 24354230

[8] Vásquez‐López F , Kreusch J , Marghoob AA . Clinical and laboratory investigations dermoscopic semiology: further insights into vascular features by screening a large spectrum of nontumoral skin lesions. Br J Dermatol. 2004;226‐231. 14996092 10.1111/j.1365-2133.2004.05753.x

[9] Zalaudek I , Kreusch J , Giacomel J , Ferrara G , Catrical C , Argenziano G . How to diagnose nonpigmented skin tumors: a review of vascular structures seen with dermoscopy: part I. Melanocytic skin tumors. J Am Acad Dermatol. 2010;63(3):361‐374. 20708469 10.1016/j.jaad.2009.11.698

[10] Lallas A , Giacomel J , Argenziano G , et al. Dermoscopy in general dermatology: practical tips for the clinician. Br J Dermatol. 2014;170(3):514‐526. 24266695 10.1111/bjd.12685

[11] Brasiello M , Zalaudek I , Ferrara G , et al. Lupus vulgaris: a new look at an old symptom ‐ The lupoma observed with dermoscopy. Dermatology. 2009;218(2):172‐174. 19060460 10.1159/000182255

[12] XX X . Dermoscopic features of cutaneous leishmaniasis: study of 52 lesions. J Am Acad Dermatol. 2017;76(6):AB97.

[13] Pellicano R , Tiodorovic‐Zivkovic D , Gourhant JY , et al. Dermoscopy of cutaneous sarcoidosis. Dermatology. 2010;221(1):51‐54. 20375489 10.1159/000284584

